# Temporary epicardial cardiac resynchronisation versus conventional right ventricular pacing after cardiac surgery: study protocol for a randomised control trial

**DOI:** 10.1186/1745-6215-13-20

**Published:** 2012-02-20

**Authors:** Stuart J Russell, Christine Tan, Peter O'Keefe, Saeed Ashraf, Afzal Zaidi, Alan G Fraser, Zaheer R Yousef

**Affiliations:** 1 Wales Heart Research Institute, Heath Park, Cardiff. CF14 4XN. UK; 2 University Hospital of Wales, Heath Park, Cardiff. CF14 4XW. UK; 3 Morriston Hospital, Swansea. SA6 6NL. UK

**Keywords:** Cardiac surgery, biventricular pacing, heart failure

## Abstract

**Background:**

Heart failure patients with stable angina, acute coronary syndromes and valvular heart disease may benefit from revascularisation and/or valve surgery. However, the mortality rate is increased- 5-30%. Biventricular pacing using temporary epicardial wires after surgery is a potential mechanism to improve cardiac function and clinical endpoints.

**Method/design:**

A multi-centred, prospective, randomised, single-blinded, intervention-control trial of temporary biventricular pacing versus standard pacing. Patients with ischaemic cardiomyopathy, valvular heart disease or both, an ejection fraction ≤ 35% and a conventional indication for cardiac surgery will be recruited from 2 cardiac centres. Baseline investigations will include: an electrocardiogram to confirm sinus rhythm and measure QRS duration; echocardiogram to evaluate left ventricular function and markers of mechanical dyssynchrony; dobutamine echocardiogram for viability and blood tests for renal function and biomarkers of myocardial injury- troponin T and brain naturetic peptide. Blood tests will be repeated at 18, 48 and 72 hours. The principal exclusions will be subjects with permanent atrial arrhythmias, permanent pacemakers, infective endocarditis or end-stage renal disease.

After surgery, temporary pacing wires will be attached to the postero-lateral wall of the left ventricle, the right atrium and right ventricle and connected to a triple chamber temporary pacemaker. Subjects will be randomised to receive either temporary biventricular pacing or standard pacing (atrial inhibited pacing or atrial-synchronous right ventricular pacing) for 48 hours.

The primary endpoint will be the duration of level 3 care. In brief, this is the requirement for invasive ventilation, multi-organ support or more than one inotrope/vasoconstrictor. Haemodynamic studies will be performed at baseline, 6, 18 and 24 hours after surgery using a pulmonary arterial catheter. Measurements will be taken in the following pacing modes: atrial inhibited; right ventricular only; atrial synchronous-right ventricular; atrial synchronous-left ventricular and biventricular pacing. Optimisation of the atrioventricular and interventricular delay will be performed in the biventricular pacing group at 18 hours. The effect of biventricular pacing on myocardial injury, post operative arrhythmias and renal function will also be quantified.

**Trial Registration:**

ClinicalTrials.gov: NCT01027299

## Background

The prevalence of heart failure is increasing throughout the industrialised world. Approximately 2-3% of the general population are diagnosed with heart failure [1] and the primary aetiology is coronary artery disease. A retrospective analysis of heart failure trials has identified at least 62% of subjects have coronary disease [2]. The total financial cost to the National Health Service is approximately £ 563 million per annum in 2006-7[3].

Modern medical therapy has substantial reduced both morbidity and mortality after a myocardial infarction. ACE inhibitors [4,5], beta blockers [6-8] and aldosterone antagonists [9,10] modulate the renin-angiotensin-aldosterone axis and neurohormonal cascade which reduces major adverse events. These medications arrest the cascade of progressive ventricular remodelling and dilatation observed in heart failure. Further ventricular remodelling can be achieved with biventricular (BiV) pacing through the reversal of electro-mechanical dyssynchrony. The reduction in ventricular volumes correlates to a reduction in heart failure events, arrhythmias and death [11,12].

Heart failure patients with stable angina and a significant burden of coronary artery disease may benefit from surgical revascularisation. However, the risk of surgery is increased and mortality rates range from 5-30% [13]. There is limited randomised control data on surgical revascularisation in subjects with severe left ventricular (LV) systolic dysfunction- ejection fraction ≤ 35%. The landmark trials of surgical revascularisation in the 1970 excluded subjects with significant LV dysfunction [14,15].

The Coronary-Artery Bypass Surgery in Patients with LV Dysfunction (STICH) trial was specifically designed to address this issue and compared optimal medical therapy to surgical revascularisation, in subjects with severe LV systolic impairment [16]. The primary endpoint of all cause mortality was not significant between the 2 groups at 56 months follow-up (41% medical v 36% surgical; p = 0.12). However, the secondary endpoint of death or cardiovascular hospitalisation was less likely in the surgical group (68% v 58%; p < 0.001).

A sub-study of the STICH trial (n = 601) also investigated the prognostic value of myocardial viability in patients with severe LV systolic impairment [17]. Viability was assessed using single-photon emission computer tomography or dobutamine echo. After adjustment for baseline variables there was no significant association between viability and mortality (p = 0.21). Neither was there a significant interaction between viability status and treatment assignment with respect to mortality (p = 0.53). However, previous meta-analysis of viability (n = 3088) reported a 79.6% reduction in annual mortality with revascularisation versus medical therapy when viable myocardium was detected (16% v 3.2%, p < 0.0001) [18]. There was no advantage of revascularisation in non-viable myocardium.

The ESC guidelines on myocardial revascularisation 2010 [13] recommend surgical revascularisation in chronic heart failure (ejection fraction ≤ 35%) in the following circumstances- Table 1:

**Table 1:** Indications for surgical revascularisation in heart failure.

Indication	Class	Level
CABG is recommended for:	I	B
• Significant left main stenosis		
• Left main equivalent		
• Proximal LAD stenosis, with 2 or 3 vessel disease		

LV aneurysmectomy during CABG is indicated in patients with a large LV aneurysm.	I	C

CABG should be considered in the presence of viable myocardium irrespective of LV end-systolic volumes.	IIa	B

CABG with ventricular reconstruction may be considered in patients with scarred LAD territory.	IIb	B

Revascularisation in the absence of myocardial viability is not recommended.	III	B

In addition, heart failure patients with unstable coronary syndromes and valvular heart disease are potential candidates for cardiac surgery. In view of the increased pre-operative risk of surgery, interest has focused on techniques including temporary BiV pacing, to optimise cardiac function and outcome in the post operative period.

BiV pacing can be performed after cardiac surgery via temporary epicardial wires attached to the right atrium, right and left ventricle. Through a mechanism of reversing electro-mechanical dyssynchrony, BiV pacing can augment stroke volume and cardiac output without increasing myocardial oxygen demand. An acute haemodynamic response has been observed in previous studies during implantation of permanent BiV pacemakers - Table 2.

**Table 2:** Trials of acute haemodynamic response to biventricular pacing.

Investigator	Study title	Sample	Result
Breithardt [26], 2003.	Acute effects of CRT on MR in systolic heart failure.	N = 24 EF ≤ 30% LBBB	Reduction in MR and improvement in dP/dT with BiV, p < 0.0001.

Leclerq [27], 1998.	Acute haemodynamic effects of BiV in patients with end-stage heart failure.	N = 18 NYHA ≥ 3 QRS ≥ 120	Increase in cardiac index BiV pacing v AAI, p < 0.001.

Aurrichio [28], 1999.	Effect of pacing chamber and AV delay on acute systolic function of paced patients with heart failure.	N = 27 NYHA ≥ 3 LBBB	Improvement in LV dP/dT with BiV pacing v DDD(RV) pacing, p < 0.01.

Kass [29], 1999.	Improved LV mechanics from acute VDD pacing in patients with DCM and conduction delay.	N = 18 QRS ≥ 120	Improvement in LV dP/dT with BiV pacing v AAI, p < 0.05.

These studies were conducted on subjects after optimising medical therapy including ACE inhibitors and beta blockers. Temporary BiV pacing after cardiac surgery has additional considerations. The haemodynamic performance of this technique maybe affected in the presence of inotropes and vasopressors, anaesthetic agents, intra-aortic balloon counter-pulsation, positive pressure ventilation and myocardial injury after circulatory arrest. In addition, ACE inhibitors are frequently discontinued prior to surgery because of post operative hypotension and renal dysfunction [19].

Previous trials of temporary BiV pacing after cardiac surgery have produced mixed results- Table 3.

**Table 3:** Acute BiV pacing studies after cardiac surgery.

Investigator	Study title	Sample	Result
Pokushalov [20], 2010.	CABG with CRT in patients with ischaemic heart failure and dyssynchrony.	N = 178 EF ≤ 35% QRS > 120	Reduced mortality with CRT, p = 0.006, reduced LOS and improved CI at 48 hours, p < 0.001.

Eberhardt [30], 2009.	BiV pacing after CABG in patients with reduced LV function.	N = 94 EF ≤ 40%	No significant difference between BiV and other pacing modes on haemodynamics or LOS.

Hanke [24], 2009.	BiV pacing after cardiopulmonary bypass in patients with reduced LV function.	N = 21 EF < 35% QRS > 120	BiV superior to DDD(RV) pacing but not DDD(LV) or AAI pacing.

Evonich [31], 2008.	Temporary BiV pacing in cardiac patients with severely reduced LV function.	N = 40 EF ≤ 30%	No significant change in LOS or haemodynamic function with BiV.

Hamad [32], 2009.	Acute haemodynamic effect of CRT in patients with poor LV function during surgery.	N = 11 QRS > 130 EF ≤ 35%	Optimised (VV) BIV pacing improved haemodynamics (p = 0.03) v RV pacing.

Muehlschlegel [33], 2008.	Temporary BiV pacing after CABG in patients with reduced ejection fraction.	N = 10 EF < 50% QRS > 120	Significant improvement in cardiac output with BiV v DDD(RV or LV).

Dzemali [34], 2008.	Impact of different pacing modes on LV function following CABG.	N = 80 EF ≤ 35%	Patients with dilated LV (mean 65 v 52mm) more likely to respond to BiV, p < 0.001.

The largest trial in this series (Pokushalov et al [20]) enrolled subjects with baseline dyssynchrony. This included a QRS duration > 120 msec or the echo criteria for dyssynchrony used in the CARE-HF trial [21]. In addition, the effect of BiV pacing was not measured in subjects with an intra-aortic balloon pump. Therefore, additional studies to assess the clinical and haemodynamic effects of BiV pacing in all subjects in sinus rhythm with severe LV systolic impairment, ejection fraction (EF) ≤ 35% irrespective of baseline dyssynchrony are indicated. This includes haemodynamic studies of BiV pacing in subjects who require intra-aortic balloon counter-pulsation and further evaluation of atrioventricular (AV) and interventricular (VV) optimisation.

## Methods/Design

### Hypothesis

• Post operative biventricular pacing may improve acute haemodynamic function after surgery by reversal of electro-mechanical dyssynchrony.

• Temporary BiV pacing may reduce the requirements for inotropic support, ventilation and renal replacement therapy and hence the duration of level 3 care.

• Optimisation of BiV pacing intervals may provide additional haemodynamic benefit compared to nominal settings.

### Study design

A multi-centred, prospective, randomised, single-blinded, intervention-control trial of patients with severely impaired LV systolic function (EF ≤ 35%) undergoing cardiac surgery for revascularisation, valve surgery or both. Inclusion and exclusion criteria are detailed in Table 4.

**Table 4:** Inclusion and exclusion criteria.

Inclusion criteria	Exclusion criteria
Age > 18 years	Permanent pacemaker or defibrillator

Sinus rhythm	Infective endocarditis

Surgical revascularisation, valve surgery or both	Hypertrophic cardiomyopathy

	Off pump cardiac surgery

	Permanent atrial arrhythmia

	Renal failure (dialysis dependent)

	Emergency revascularisation.

The trial was approved by the 'Research and Development' departments, at the University Hospital of Wales, Cardiff (09/CAD/4628-sponsor) and Morriston Hospital, Swansea, UK (S10CardS961). The trial has been approved by the Dyfred Powys Research Ethics Committee-09/WMW01/27. The trial will be conducted in accordance with the 'Declaration of Helsinki' and all subjects will provide written consent. This trial is registered with clinicaltrials.gov NCT01027299.

### Pre-operation

Patients will be invited to participate in the trial if they are awaiting cardiac surgery. This includes elective patients and in-house referrals scheduled for revascularisation, valve surgery or both. Subjects with a recent history of an acute coronary syndrome or episode of decompensated heart failure (< 30 days) will be included. However, unstable subjects with medically refractory angina and/or pulmonary oedema requiring emergency surgery will be excluded from the trial.

After enrolment an ECG will be performed to confirm sinus rhythm and measure QRS duration and a transthoracic echocardiogram to measure ejection fraction using the Simpson's bi-plane method of discs. A detailed echo study will be performed (GE, Vivid 7, Horten, Norway), to evaluate LV systolic and diastolic function, valve function and cardiac dyssynchrony- Table 5. Subjects with EF > 35% will exit the trial.

**Table 5:** Echo protocol.

Echo window	Modality	Measurement
Parasternal long axis	MMode	LV internal dimensions
		LV wall thickness
		Septum to lateral wall delay
	Colour flow	Mitral regurgitation

Parasternal short axis	PW doppler	RVOT- pulmonary pre-ejection interval (PPEI)
	CW doppler	PA acceleration time

Apical 4 chamber	2D	LA and RA area
		LVEDV and LVESV
	MMode	Long axis displacement- RV lateral/LV septal and
		lateral walls
	Colour flow	Mitral regurgitation
	PW doppler	Mitral inflow
		LVOT- aortic pre-ejection interval (APEI)
		IVRT
	MVI	Medial and lateral E'
		Basal segments- peak velocity.

Apical 2 chamber	2D	LVEDV and LVESV
	MVI	Basal segments- peak velocity.

Apical 3 chamber	MVI	Basal segments- peak velocity.

Echo measurements of dyssynchrony included:

• Interventricular mechanical delay (= APEI-PPEI)

• Atrio-ventricular delay (APEI)

• Time-delay to peak systolic velocity in the basal segments of the septal and lateral wall using myocardial velocity imaging (MVI) Q analysis.

A repeat transthoracic echo will be performed after discharge from the cardiac intensive care unit (CITU) between day 3 and 5 post surgery.

Baseline blood tests will be sent to measure:

• Full blood count (Haemoglobin, haematocrit and mean cell volume)

• Creatinine (eGFR calculation)

• TnT: Troponin T

• BNP: Brain naturetic peptide

• NGAL: Neutrophil gelatinase associated lipocalin- renal injury

Blood samples will be taken at baseline, 18 hours, 48 hours and 72 hours after admission to CITU.

An assessment of myocardial viability using dobutamine stress echo (GE, Vivid 7, Horten, Norway) will be considered for each patient. Each case will be discussed with the cardiac surgeon before this test. Principal exclusions will be a recent acute coronary syndrome or episode of decompensated heart failure, valvular heart disease, prior viability assessment or physician preference. Viability assessment will be performed by an experienced operator with ECG and blood pressure monitoring. Dobutamine will be initially infused at a rate of 5 microg/kg/min and increased in steps to 10, 20, 30 and 40 micrograms every 3 minutes. Atropine 0.25 mg will be added as a bolus at intervals of 1 minute up to a total dose of 1 mg. The maximum target heart rate = [(220-age) × 0.85]. Viability will be defined as 5 or more segments with abnormal resting function but manifesting contractile reserve during dobutamine testing [17].

### Randomisation

Randomisation into 2 groups will be performed after induction of anaesthesia using commercially available software to minimise for confounding variables -Table 6. Group 1 will be assigned to BiV pacing and group 2 to standard pacing. Standard pacing will be atrial inhibited pacing (AAI) unless right ventricular pacing is required, then atrial-synchronous right ventricular pacing (DDD-RV) is indicated. The intended duration of pacing will be 48 hours, but could be modified by the attending physician.

**Table 6:** Variables considered at the time of randomisation.

Parameter	Reference	
LV size	≤ 60 mm	> 60 mm

QRS duration	≤ 120 msec	> 120 msec

Euroscore	≤ 10	> 10

Intra-aortic balloon pump	No	Yes

Valve surgery	No	Yes

Ejection fraction	≤ 20%	> 20%

### Operation

All patients will receive standard monitoring with ECG, pulse oximetry, a radial arterial line with cardiac output monitor attached to a Vigileo monitor (Flotrac MHD8R, Edwards Lifesciences, Dominican Rep). Central line and a pulmonary arterial catheter (either 7.5F CCO: 139HF75P or 7F TD: 131 F7, Edwards Lifesciences, Irvine, USA) will be inserted after intravenous induction of general anaesthesia. The techniques of anaesthesia and cardio-pulmonary bypass (CPB) will be standardized. After premedication with benzodiazepines, anaesthesia will be induced with iv propofol (1-2 mg/kg) and fentanyl (10-20 μg/kg); muscle relaxation will be achieved with rocuronium (1 mg/kg), vecuronium (0.2 mg/kg) or pancuronium bromide (0.1 - 0.2 mg/kg). The airway will be intubated and mechanically ventilated with oxygen, air and isoflurane 1-2%. Propofol and remifentanil infusion may be used in addition to isoflurane for maintenance of anaesthesia.

Intravenous fluids (Hartmann' s solution or gelofusin), vasopressors (metaraminol or phenylephrine) and inotropes (enoximone or milirone, dopamine, noradrenaline and adrenaline etc.) will be titrated according to patients' haemodynamic status. Prophylactic antibiotics (cefuroxime and teicloplanin) and antifibrinolytic agents such as tranexamic acid (2 g) will be administered prior to sternotomy.

Operative monitoring will be identical in all patients. The extracorporeal circuit will comprise a Stockert roller pump (Shiley Inc, Irvine, CA), a Shiley S100 membrane oxygenator, and polyvinylchloride tubes. Patients will be given heparin just before the institution of CPB with 300 IU/kg, with additional dosing as necessary to maintain the activated clotting time greater than 480 seconds. Non-pulsatile extracorporeal circulation will be initiated at flows of 2.4 to 2.6 L m^-2 ^min^-1^. Moderate systemic hypothermia (32°C nasopharyngeal) will be uniformly used. Cardiac arrest will be achieved by infusion of 1 L of cold blood cardioplegic solution and topical slush.

All distal anastomoses will be performed during a single period of cross clamping, and the proximal anastomoses to the aorta will be completed during the re-warming period. Extracorporeal circulation will be terminated at a nasopharyngeal temperature of 37°C. Heparin will be neutralized after the end of CPB with protamine sulphate (1 mg/100 IU heparin).

Before weaning from the cardio-pulmonary bypass circuit, temporary unipolar epicardial pacing wires will be sutured to the roof of the right atrium, the right ventricular outflow tract and the postero-lateral free wall of the left ventricle (first obtuse marginal territory) avoiding scar tissue. There will be a total of 6 wires, 3 active and 3 indifferent wires. The wires will be attached via extension cables to a temporary external triple chamber pacemaker (Osypka PACE 300, Germany).

Pacing will be commenced prior to separation from the bypass circuit according to each patient's randomisation. Subjects in the BiV group received DDD-BiV pacing, base rate 90 bpm, AV interal 120 msec, max tracking rate 110 bpm, VV interval -5 msec (-ve indicating LV pre-activation). The standard pacing group received AAI pacing at base rate 90 bpm unless ventricular pacing will be indicated. In this situation DDD-RV pacing will be programmed, base rate 90 bpm, AV interval 150 msec and max tracking rate 110 bpm. RV pacing will be kept to a minimum.

Inotropes including, enoximone or milrinone in combination with noradrenaline will be administered according to haemodynamic requirements. The target range for haemodynamic parameters after surgery:

• Mean arterial pressure 60-70 mm Hg

• Pulmonary capillary wedge pressure < 18 mm Hg

• Cardiac index 2.0-2.5 L/min/m^2^

### Haemodynamic studies

Haemodynamic studies will be performed after admission to the CITU after surgery (time = 0). The first study will be performed using a pulmonary arterial (PA) catheter, once the subject is euvolaemic and has a stable inotropic requirement. Cardiac output/index, pulmonary artery pressures, pulmonary capillary wedge pressure, central venous pressure, mean arterial pressure (MAP) and LV stroke work index will be recorded.

For thermodilution measurements, 3-5 sets of cardiac output measurements will be obtained to ensure consistent results. Measurements will be taken in the following pacing modes: atrial inhibited (AAI), right ventricle only (VVI), dual chamber (DDD-RV) and (DDD-LV) and BiV pacing. A minimum of 5 minutes will be allowed between changing the pacing mode and obtaining haemodynamic data. A pacing mode will be discontinued if it causes > 10 mm Hg drop in MAP. The base rate of pacing will be set at 10 beats above intrinsic rhythm up to a maximum heart rate of 110 bpm. The pacing modes will be selected at random. The studies will be repeated at 6 hours, 18 hours and 24 hours. After the 24 hour measurements the PA catheter will be removed. Cardiac output data will be also obtained from the radial pulse contour analysis (FLOTRAC) monitor at the same time points as the PA catheter, except for subjects with an intra-aortic balloon pump.

Optimisation of the atrio-ventricular (AV) and interventricular (VV) delays will be performed at 18 hours. A range of AV delays from 80-180 msec in 20 msec intervals and then VV delays from -40 to 40 msec (-ve indicating LV pre-activation) in 20 msec intervals will be performed. The optimal AV and VV delays will be programmed for patients assigned to BiV pacing.

### Endpoints

The primary endpoint of the trial is the duration of level 3 care [22] required after cardiac surgery. Level 3 care is defined as the duration of invasive mechanical ventilation or multiple organ support or multiple inotropes/single inotrope and intra-aortic balloon pump. The majority of patients would reach the primary endpoint after extubation and then once weaned down to a single inotrope. In the cases where inotropes are restarted, the endpoint is taken to be the final transition from level 3 to level 2 care before discharge from the intensive care unit.

Secondary endpoints are:

• Cardiac index at 18 hours (AAI v BiV).

• Duration and total dose of inotropes/intra aortic balloon counter-pulsation.

• Renal function and requirement for haemofiltration.

• Arrhythmia after surgery until hospital discharge.

• Change in brain naturetic peptide, troponin T and neutrophil gelatinase associated lipocalin.

• Myocardial infarction, re-intubation or re-sternotomy.

• 30 day all cause mortality.

Subjects will complete the trial 30 days after the operation.

### Statistical Analysis

No previous studies have investigated the effect of BiV pacing on the duration of level 3 care after cardiac surgery; previous studies have reported the total duration of CITU stay.

Pokushalov et al [20] reported an average CITU stay of 3.9 ± 0.6 days in the subjects after cardiac surgery, compared with 2.5 ± 0.5 days in subjects who had BiV pacing after cardiac surgery - a 36% reduction. We anticipate that the duration of level 3 care will be shorter than the duration of CITU stay, perhaps with less variance, but we have used these mean values and standard deviations for our power calculation.

Using the standard formula described by Campbell and Matchin [23]:

$$\text{Sample}\;\text{size}=\frac{2\times {\left({\text{Z}}_{1\alpha }+{\text{Z}}_{2\beta }\right)}^{2}\times \sigma^{2}}{\delta^{2}}$$

σ = standard deviation of the population

δ = anticipated benefit from the intervention

(Z_1α _+ Z_2β_) = normal distribution of function

(1-β) = 80% and α = 5%.

From standard tables (Z_1α _+ Z_2β_) ^2 ^= 7.85

$$\text{Sample}\;\text{size}=\frac{2\times \left(7.85\right)\times 0.6^{2}}{{\left(0.15\times 3.9\right)}^{2}}=\frac{5.652}{0.342}=16.5$$

In order to demonstrate a 15% reduction in the duration of level 3 care, with power 80% and with p < 0.05 for a two-sided test, the estimated total sample size required is 34 subjects.

For haemodynamic comparison of paired data at 18 hours to compare BiV pacing to standard (AAI) pacing, Hanke et al [24] observed a cardiac index of 2.7 ± 0.6 L/min/ml^2 ^in the standard pacing group. Allowing a 0.3 L/min/ml^2 ^(11%) improvement in cardiac index with BiV pacing, significance 5% and power 80%, a sample size of 32 subjects would be required.

Considering both of these previous studies, the minimum number of subjects who will be recruited and randomised into this study, will be 34; allowing for a 1/3 drop-out rate, the minimum number to be recruited will be 45. In the 2 cardiac surgical centres in which this study will be performed the proportions of all patients undergoing cardiac surgery who have impaired LV function and sinus rhythm, and who would therefore be eligible for the study are 2% and 8% (approximately 65 patients in a total of 1414 operations per year); this study should therefore be feasible within a maximum of 2 years.

Data obtained for the primary endpoint and haemodynamic studies will be analysed for normality using the Kolmogorov-Smirnov test. Normally distributed variables will be analysed using unpaired or paired Student's T tests respectively. Non-parametric data will be analysed using the Mann-Whitney U or Kruskal-Wallis test. If continuous variables are not matched between intervention and control groups at baseline, inter-group comparisons will be made using an analysis of covariance with adjustments made for baseline differences. Changes in proportions for categorical data will be analysed with the Chi-square test or Fisher's exact test.

Analysis will be intention-to-treat and a p-value of < 0.05 will be considered statistically significant.

### Monitoring and safety

All adverse events, including serious adverse events (SAE), will be recorded and followed up for the duration of the study or until resolution. Assessment of adverse events will be performed by study investigators. All SAEs will be graded and reported to the sponsor. Any suspected unexpected serious adverse reactions will be reported to the sponsor and ethics committee.

### Sub-study- control subjects

The beneficial effects of biventricular pacing are related to the reversal of dyssynchrony. The prevalence of dyssynchrony in patients with preserved LV function awaiting cardiac surgery and the effect of bypass surgery on indices of dyssynchrony requires further evaluation.

Therefore, patients in sinus rhythm, EF ≥ 45% and awaiting revascularisation will be invited into the sub-study. A transthoracic echocardiogram will be performed (Table 5) before and after surgery, on day 3-5. The data will be analysed for acute changes in mechanical dyssynchrony after on-pump surgery.

Wang et al [25] measured the interventricular delay in hypertensive subjects with preserved LV function- 21 msec ± 19. Using a 5% significance level and 80% power, to detect a 20 msec change in IVMD, a sample size of 8 subjects is needed.

### Summary

This trial is designed to assess the clinical and haemodynamic response to temporary BiV pacing after cardiac surgery. All subjects in sinus rhythm with severe LV systolic impairment (EF ≤ 35%) will be enrolled, irrespective of QRS duration or pre-operative requirement for intra aortic balloon counter pulsation.

A reduction in the length of level 3 care, acute haemodynamic optimisation or protection against arrhythmias or renal dysfunction/haemofiltration with BiV pacing would be a clinically significant finding.

### Trial status

The trial started recruitment in February 2009 and we have recruited 31 patients.

## List of abbreviations

AAI: Atrial inhibited pacing; APEI: Aortic pre-ejection interval; AV: Atrio-ventricular; BiV: Biventricular; BNP: Brain naturetic peptide; BPM: Beats per minute; CABG: Coronary artery bypass surgery; CCO: Continuous cardiac output; CI: Cardiac index; CITU: Cardiac intensive care unit; CRT: Cardiac resynchronisation therapy; CW: Continuous wave Doppler; DCM: Dilated cardiomyopathy; DDD: Dual chamber pacing; EC: Electrocardiogram; eGRF: Estimated glomerular filtration rate; EF: Ejection fraction; IVRT: Isovolaemic relaxation time; LA: Left atrium; LAD: Left anterior descending coronary artery; LOS: Length of stay; LV: Left ventricle; LVEDV/LVESV: Left ventricular end-diastolic volume/left ventricular end-systolic volume; MAP: Mean arterial pressure; MR: Mitral regurgitation; MVI: Myocardial velocity imaging; NGAL: Neutrophil gelatinase associated lipocalin; PA: Pulmonary artery; PPEI: Pulmonary pre-ejection interval; PW: Pulse wave Doppler; RA: Right atrium; RV: Right ventricle; RVOT: Right ventricular outflow tract; SAE: Serious adverse event; TD: Thermodilution; TnT: Troponin T; VV: Interventricular; VVI: Ventricular inhibited pacing

## Competing interests

The sponsor form this trial is the Cardiff and Vale Local Heath Board (formerly the University of Wales NHS Trust) Cardiff, Wales, UK.

## Authors' contributions

All authors have been involved in the design of the study and the drafting of the manuscript and will participate in patient recruitment and. SJR will collect the data including haemodynamic measurements using the pulmonary arterial catheter and the trial transthoracic echocardiograms. AGF will perform the dobutamine viability studies. All authors will be involved in the statistical analysis and data interpretation. All authors have read and approved the final manuscript.

## Authors' information

SJR is a Cardiology Research Fellow.
